# Bactericidal efficacy of meropenem in combination with cefmetazole against IMP-producing carbapenem-resistant *Enterobacteriaceae*

**DOI:** 10.1186/s13104-019-4779-x

**Published:** 2019-11-09

**Authors:** Ryuichiro Abe, Hideharu Hagiya, Yukihiro Akeda, Norihisa Yamamoto, Yoshikazu Ishii, Kazunori Tomono

**Affiliations:** 1 Department of Infection Control and Prevention, Osaka University Graduate School of Medicine, 2-15 Yamadaoka, Suita, Osaka 565-0871 Japan; 2 Research Institute for Microbial Diseases, Osaka University, Osaka, Japan; 3 Department of Anesthesiology and Intensive Care Medicine, Graduate School of Medicine, Osaka University, Osaka, Japan; 4 Department of General Medicine, Graduate School of Medicine Dentistry, and Pharmaceutical Sciences, Okayama University, Okayama, Japan; 5 Department of Microbiology and Infectious Diseases, Toho University School of Medicine, Tokyo, Japan

**Keywords:** Carbapenem-resistant *Enterobacteriaceae*, Carbapenemase-producing *Enterobacteriacea*, Cephamycin, Cefmetazole, IMP

## Abstract

**Objective:**

Carbapenem-resistant *Enterobacteriaceae* (CRE) are among the most severe threats to public and clinical health because of their high levels of resistance to various antibiotics. We assessed the efficacy of combination therapy with meropenem (MEM) and cefmetazole (CMZ) against Imipenemase (IMP)-producing CRE, using the checkerboard method and time-killing assay on 13 *Enterobacteriaceae* isolates harboring *bla*_IMP-1_ (4 *Enterobacter hormaechei*, 5 *Escherichia coli*, and 4 *Klebsiella pneumoniae* isolates) and 13 isolates harboring *bla*_IMP-6_ (8 *E. coli* and 5 *K. pneumoniae* isolates).

**Results:**

Minimum inhibitory concentrations (MICs) of MEM and CMZ ranged from 2 to 64 and 64 to 2048 μg/mL, respectively. Checkerboard method demonstrated the synergy of the MEM/CMZ combination in all the tested IMP-producing CRE isolates, and the time-kill assay indicated a bactericidal effect for both *bla*_IMP-1_ and *bla*_IMP-6_ positive CRE when MEM/CMZ combination was used. In vitro, the MEM/CMZ combination was potentially effective against IMP-1- or IMP-6-producing CRE. Further investigations including in vivo animal studies and clinical studies are warranted to corroborate the clinical utility of the novel combination therapy.

## Introduction

Carbapenems are the last-resort antibiotics for the treatment of various infections caused by multidrug-resistant gram-negative bacteria. The wide global dissemination of carbapenem-resistant *Enterobacteriaceae* (CRE) is a serious threat to global public health and is a major concern to clinicians. Carbapenem resistance is mainly due to the production of carbapenemases whose genes are encoded on plasmids that are transmitted easily across bacterial species, which has resulted in the rapid spread of CRE worldwide [1].

Carbapenemases are categorized into mainly three classes (classes A, B, and D) of the Amber β lactamase classification. The class A *Klebsiella pneumoniae* carbapenemases (KPC) are one of the most prevalent carbapenamases. The class D Oxacillin-hydrolysing (OXA)-48 carbapenemase producers have disseminated globally. Class B metallo-β-lactamases (MBLs) mainly comprise the Verona Integron-Mediated (VIM)-, Imipenemase (IMP)-, and New Delhi Metallo-beta-lactamase (NDM)-types. Of these, NDM producers have rapidly spread worldwide since the first reports of their emergence in 2009 [1]. Despite their global dissemination, KPCs, OXAs, and NDMs are still not common in Japan, where IMP-1 and IMP-6 are the exclusively predominant carbapenemases [2, 3].

Treatment options for patients infected with carbapenemase-producing *Enterobacteriaceae* (CPE) are limited. For class A carbapenemases, such as KPC, a double-carbapenem therapy has been used [4]. Additionally, ceftazidime/avibactam combination, which is officially approved in Europe and the United States but not in Japan, has exhibited potent activity against OXA-48 producing gram-negative organisms [5]. However, MBL producers are resistant to these new treatments, and few studies have explored treatment strategies for MBL-producing *Enterobacteriaceae* [5, 6].

In a previous study, we demonstrated the in vitro efficacy of the combination of meropenem (MEM) and cefmetazole (CMZ) against KPC producers [7]. In this study, we investigated the in vitro activity of the MEM/CMZ combination therapy against IMP-producing CRE.

## Main text

### Methods

We used 13 *Enterobacteriaceae* isolates harboring *bla*_IMP-1_ (4 *Enterobacter hormaechei*, 5 *Escherichia coli*, and 4 *Klebsiella pneumoniae*) provided by Toho University, and 13 *Enterobacteriaceae* isolates harboring *bla*_IMP-6_ (8 *E. coli* and 5 *K. pneumoniae*) obtained in our previous CRE surveillance in Osaka, Japan [2]. *E. hormaechei* isolates belonging to *Enterobacter cloacae* complex were included in this analysis as one of *bla*_IMP-1_ representative carriers, which were detected in an outbreak reported in Tokyo, Japan [3]. Through the experiments explained below, we used meropenem trihydrate (Tokyo Chemical Industry, Japan, Tokyo) and cefmetazole sodium salt (Sigma-Aldrich, Saint Louis, MO, United States) as antibiotic agents.

We determined minimum inhibitory concentrations (MICs) of MEM and CMZ by the broth microdilution method based on the Clinical and Laboratory Standards Institute (CLSI) document M07-A10 [8]. Bacterial growth was evaluated by visible observation for turbidity. Briefly, we inoculated 5×10^5^ colony forming units (CFU)/mL of bacterial suspension into cation-adjusted BBL™ Mueller–Hinton II Broth (Becton, Dickinson and Company, Sparks, MD, USA) and incubated at 35 °C in ambient air for 18 h. MIC was defined as the lowest concentration of the tested antimicrobial that completely inhibited the growth of bacteria.

Details of the checkerboard method and time-killing assay have been presented previously [7]. In the checkerboard method, synergistic effect between MEM and CMZ against IMP-1 or IMP-6 producers was quantified by calculating the fractional inhibitory concentration (FIC) index. FIC index value ≤ 0.5 was defined as synergy, > 0.5 to ≤ 4.0 as indifferent, and > 4.0 as antagonistic. The assay was performed in duplicate. In case a synergistic effect was observed, MEM MIC fold-reduction was determined based on the lowest FIC index.

We conducted time-killing assays using the following two *E. coli* isolates: TUM13773 carrying *bla*_IMP-1_ and E109 carrying *bla*_IMP-6_. During these assays, each isolate was incubated in Muller-Hinton II broth devoid of antibiotic (control) and with MEM, CMZ, or MEM/CMZ combination at the concentration of 25% of the MIC for individual isolates. CFUs of bacteria at 3, 6, 9, and 24 h after beginning the treatment were counted. The averages of CFUs were calculated from duplicated assays. We defined an efficacy of the combination therapy as bactericidal when ≥ 3 log_10_ CFU/mL reduction compared to the initial bacterial count was observed.

## Results

MIC ranges of MEM and CMZ for isolates harboring *bla*_IMP-1_ were 2 to 8 μg/mL and 64 to 2048 μg/mL, respectively. Similarly, those harboring *bla*_IMP-6_ ranged from 16 to 32 μg/mL and from 64 to 1024 μg/mL, respectively (Table 1). No isolates were susceptible to both MEM and CMZ based on the CLSI breakpoint values [9].

**Table 1:** Minimum inhibitory concentrations (MICs) and fractional inhibitory concentration (FIC) index of *bla*_IMP-1_ or *bla*_IMP-6_ positive *Enterobacteriaceae* isolates

	Isolates		MIC (μg/mL)		MEM MIC reduction	FIC index	Evaluation
			MEM	CMZ			
IMP-1 producing isolates	TUM10695	*E. hormaechei*	2	512	1/4, 1/8	0.27, 0.19	Synergy
	TUM11051	*E. hormaechei*	8	2048	1/4, 1/4	0.50, 0.50	Synergy
	TUM11052	*E. hormaechei*	8	1024	1/4, 1/4	0.28, 0.27	Synergy
	TUM11134	*E. hormaechei*	2	1024	1/4, 1/4	0.31, 0.50	Synergy
	TUM11259	*E. coli*	8	512	1/8, 1/8	0.25, 0.38	Synergy
	TUM13773	*E. coli*	4	256	1/4, 1/8	0.50, 0.37	Synergy
	TUM14683	*E. coli*	4	512	1/16, 1/8	0.19, 0.19	Synergy
	TUM14697	*E. coli*	2	64	1/4, 1/8	0.38, 0.19	Synergy
	TUM14759	*E. coli*	4	512	1/8, 1/4	0.25, 0.28	Synergy
	TUM13774	*K. pneumoniae*	8	256	1/8, 1/8	0.25, 0.38	Synergy
	TUM13775	*K. pneumoniae*	8	512	1/8, 1/8	0.25, 0.25	Synergy
	TUM14366	*K. pneumoniae*	8	256	1/8, 1/4	0.25, 0.31	Synergy
	TUM14380	*K. pneumoniae*	2	512	1/4, 1/4	0.50, 0.38	Synergy
IMP-6 producing isolates	E015	*E. coli*	16	256	1/8, 1/16	0.16, 0.13	Synergy
	E020	*E. coli*	16	256	1/8, 1/16	0.13, 0.19	Synergy
	E030	*E. coli*	32	512	1/16, 1/16	0.09, 0.08	Synergy
	E038	*E. coli*	16	512	1/32, 1/16	0.05, 0.14	Synergy
	E046	*E. coli*	32	1024	1/8, 1/8	0.14, 0.14	Synergy
	E070	*E. coli*	16	256	1/32, 1/32	0.19, 0.06	Synergy
	E109	*E. coli*	16	64	1/32, 1/32	0.28, 0.28	Synergy
	E138	*E. coli*	64	1024	1/8, 1/8	0.19, 0.19	Synergy
	E039	*K. pneumoniae*	32	256	1/16, 1/16	0.13, 0.13	Synergy
	E045	*K. pneumoniae*	32	256	1/8, 1/16	0.16, 0.09	Synergy
	E065	*K. pneumoniae*	32	256	1/32, 1/32	0.09, 0.06	Synergy
	E085	*K. pneumoniae*	32	512	1/8, 1/8	0.19, 0.16	Synergy
	E139	*K. pneumoniae*	32	1024	1/8, 1/4	0.25, 0.27	Synergy

*CMZ* cefmetazole, *MEM* meropenem

FIC index was measured in duplication. FIC index was calculated in following formula; FIC index = (MIC of MEM measured in combination with CMZ)/(MIC of MEM only) + (MIC of CMZ measured in combination with MEM)/(MIC of CMZ only). FIC index ≤ 0.5 was defined as synergy, > 0.5 to ≤ 4.0 as indifferent, and > 4.0 as antagonistic. MEM MIC fold-reduction by CMZ was calculated at the lowest FIC index

The checkerboard method showed that the combination of MEM and CMZ was synergistically active against all the tested IMP-producing CRE isolates (Table 1). MEM MICs in the combination achieved 4- to 32-fold reduction of the MIC of MEM alone.

**Fig. 1:**
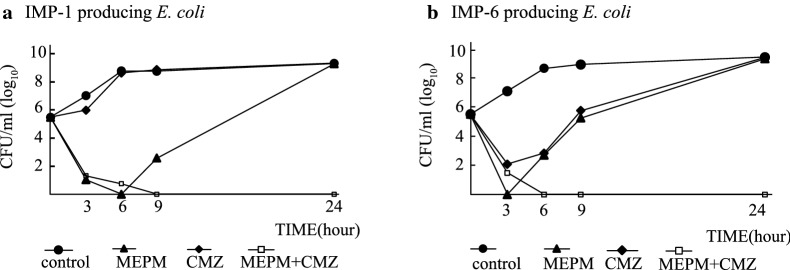
Time kill curves for IMP-1- or IMP-6-producing *E. coli* isolates with MEM or CMZ only, or both MEM and CMZ. *E. coli* isolate TUM13773 carrying *bla*_IMP-1._ **a** and *E. coli* isolate E109 carrying *bla*_IMP-6_. **b** were incubated in Mueller–Hinton II Broth (black circle) or supplemented with MEM (black up-pointing triangle), with CMZ (black diamond) or with combination of MEM and CMZ (square) at 37 °C. All the antibiotics were used at concentrations of 25% of their MIC for individual isolates. Viable cells per milliliter before incubation and after incubation for 3, 6, 9, and 24 h were counted on Muller Hinton II agar plates after overnight incubation at 37 °C. Assays were performed in duplicate and the logarithm of the average CFU/mL was plotted

In the time-killing assays, the viable bacteria treated with each antibiotic alone regrew at 24 h (Fig. 1). Contrarily, upon using the combination of 0.25 × MIC MEM and 0.25 × MIC CMZ, no growth was detected at 9 h and 24 h, indicating a bactericidal effect against the tested isolates.

### Discussion

We herein demonstrated the in vitro efficacy of MEM in combination with CMZ against IMP-producing CRE isolates. In addition to the double-carbapenem therapy [4] and ceftazidime/avibactam [5], newer β-lactamase inhibitors including relebactam (MK-7655), nacubactam (OP0595), zidebactam (WCK 5107), and vaborbactam (RPX7009) have been developed to treat CPE that produce serine-carbapenemases [10]. On the other hand, there is less available treatment option for infections caused by MBL-producing CRE.

Still much remains to be done, this investigation is very novel to the literature, in that it was intended to corroborate the potency of CMZ to cancel the function of IMP-type carbapenemases. CMZ is one of the established antimicrobials with accumulated data of its use. It is a cephamycin antibiotic that is stable against extended-spectrum β-lactamases (ESBLs) [11], but is hydrolyzed by carbapenemases, including IMPs [12]. Presently, CRE isolates that produced IMPs were highly resistant to CMZ, with MICs ranging 64 to 2048 µg/mL. These findings may indicate a high affinity of CMZ to IMP-type carbapenemases, and is consistent with our hypothesis that CMZ binds avidly to the carbapenemases, helping MEM exert its bactericidal activity, as seen in double-carbapenem therapy [4]. To demonstrate the superiority of CMZ to MEM in terms of affinity to IMP-type carbapenemases, we need to perform a kinetic assay in future study. ESBL producing *Enterobacteriaceae* have globally disseminated [12] and CRE isolates co-harboring genes encoding ESBLs and carbapenemases have been previously described [13, 14]. Particularly, *bla*_IMP-6_ was reported to disseminate mainly through the horizontal transmission of the prevalent plasmid, pKPI-6, which simultaneously carries *bla*_IMP-6_ and *bla*_CTX-M2_ [15]. The MEM and CMZ combination therapy would be a ready-to-apply, cost-effective strategy for IMP-producing CRE even against producing ESBLs, compared to the newer β-lactamase inhibitors.

## Limitations

However, several limitations remain to be overcome prior to clinical use. First, the CMZ concentrations tested in this study exceed the serum levels attainable in humans. Second, as shown in our previous study [7], an inoculum effect may exist in this combination therapy, possibly resulting in treatment failure using a high bacterial inoculum. Third, various antimicrobial resistance mechanisms, such as AmpC-type beta-lactamases, efflux pumps, and porin loss, may influence the inhibitory activity of CMZ.

Despite these limitations, the collective data from this study demonstrate a preferential effect of MEM and CMZ when used in combination against IMP-producing CRE in vitro. Faced with the limited availability of new antimicrobials, the revived use of an existing antimicrobial agent could provide effective treatment. In vivo experiments as well as pharmacokinetic and pharmacodynamic studies are required before the clinical application of the new combination therapy.

## Data Availability

Not applicable.

## Abbreviations

CFU: colony forming units
CLSI: Clinical and Laboratory Standards Institute
CMZ: cefmetazole
CPE: carbapenemase-producing *Enterobacteriaceae*
CRE: carbapenem-resistant *Enterobacteriaceae*
ESBLs: extended-spectrum β-lactamases
FIC: fractional inhibitory concentration
IMP: imipenemase
KPC: *Klebsiella pneumoniae* carbapenemases
MBL: metallo-β-lactamases
MEM: meropenem
MICs: minimum inhibitory concentrations
NDM: New Delhi Metallo-beta-lactamase
OXA: oxacillin-hydrolysing
VIM: Verona Integron-Mediated

## References

[1] Logan LK, Weinstein RA (2017). The epidemiology of carbapenem-resistant enterobacteriaceae: the impact and evolution of a global menace. J Infect Dis.

[2] Yamamoto N, Asada R, Kawahara R, Hagiya H, Akeda Y, Shanmugakani RK (2017). Prevalence of, and risk factors for, carriage of carbapenem-resistant *Enterobacteriaceae* among hospitalized patients in Japan. J Hosp Infect.

[3] Aoki K, Harada S, Yahara K, Ishii Y, Motooka D, Nakamura S (2018). Molecular characterization of IMP-1-producing enterobacter cloacae complex isolates in Tokyo. Antimicrob Agents Chemother.

[4] Giamarellou H, Galani L, Baziaka F, Karaiskos I (2013). Effectiveness of a double-carbapenem regimen for infections in humans due to carbapenemase-producing pandrug-resistant *Klebsiella pneumoniae*. Antimicrob Agents Chemother..

[5] Kazmierczak KM, de Jonge BLM, Stone GG, Sahm DF (2018). In vitro activity of ceftazidime/avibactam against isolates of *Enterobacteriaceae* collected in European countries: INFORM global surveillance 2012–15. J Antimicrob Chemother..

[6] Tangden T, Hickman RA, Forsberg P, Lagerback P, Giske CG, Cars O (2014). Evaluation of double- and triple-antibiotic combinations for VIM- and NDM-producing *Klebsiella pneumoniae* by in vitro time-kill experiments. Antimicrob Agents Chemother..

[7] Hagiya H, Aoki K, Akeda Y, Yamamoto N, Shanmugakani RK, Ishii Y (2019). In vitro effectiveness of meropenem and cefmetazole combination treatment against KPC-2-producing *Enterobacteriaceae*. Microb Drug Resist.

[8] Clinical and Laboratory Standards Institute (CLSI) (2015). M07-A10. Methods for dilution antimicrobial susceptibility tests for bacteria that grow aerobically: approved standards.

[9] Clinical and Laboratory Standards Institute (2018). M100-S28. Performance standards for antimicrobial susceptibility testing.

[10] Bowers DR, Huang V (2016). Emerging issues and treatment strategies in carbapenem-resistant *Enterobacteriaceae* (CRE). Curr Infect Dis Rep.

[11] Doi A, Shimada T, Harada S, Iwata K, Kamiya T (2013). The efficacy of cefmetazole against pyelonephritis caused by extended-spectrum beta-lactamase-producing *Enterobacteriaceae*. Int J Infect Dis.

[12] Jacoby GA, Munoz-Price LS (2005). The new beta-lactamases. N Eng J Med.

[13] Huang W, Wang G, Sebra R, Zhuge J, Yin C, Aguero-Rosenfeld ME (2017). Emergence and evolution of multidrug-resistant *Klebsiella pneumoniae* with both *bla*_KPC_ and *bla*_CTX-M_ integrated in the chromosome. Antimicrob Agents Chemother..

[14] Rojas LJ, Weinstock GM, De La Cadena E, Diaz L, Rios R, Hanson BM, Brown JS (2017). An analysis of the epidemic of *Klebsiella pneumoniae* carbapenemase-producing *K. pneumoniae*: convergence of two evolutionary mechanisms creates the “Perfect Storm”. J Infect Dis.

[15] Kayama S, Shigemoto N, Kuwahara R, Oshima K, Hirakawa H, Hisatsune J (2015). Complete nucleotide sequence of the IncN plasmid encoding IMP-6 and CTX-M-2 from emerging carbapenem-resistant *Enterobacteriaceae* in Japan. Antimicrob Agents Chemother..

